# Supplementary material for: The Psu protein of phage satellite P4 inhibits transcription termination factor ρ by forced hyper-oligomerization
Source: Nat Commun. 2025 Jan 9;16:550. doi: 10.1038/s41467-025-55897-9 (PMC11718236; doi:10.1038/s41467-025-55897-9)

## Supplementary Information

# The Psu protein of phage satellite P4 inhibits transcription termination factor ρ by forced hyper-oligomerization

Daniela Gjorgjevikj[1,#], Naveen Kumar[2], Bing Wang[3], Tarek Hilal[1,4], Nelly Said[1], Bernhard Loll[1], Irina Artsimovitch[3], Ranjan Sen[2], Markus C. Wahl[1,5,*]

[1] Freie Universität Berlin, Institute of Chemistry and Biochemistry, Laboratory of Structural Biochemistry, Takustr. 6, D-14195 Berlin, Germany

[2] Laboratory of Transcription, Centre for DNA Fingerprinting and Diagnostics, Inner Ring Road, Uppal, Hyderabad-500039, India

[3] Department of Microbiology and Center for RNA Biology, The Ohio State University, Columbus, OH, USA

[4] Freie Universität Berlin, Institute of Chemistry and Biochemistry, Research Center of Electron Microscopy and Core Facility BioSupraMol, Fabeckstr. 36a, 14195 Berlin, Germany

[5] Helmholtz-Zentrum Berlin für Materialien und Energie, Macromolecular Crystallography, Albert-Einstein-Str. 15, D-12489 Berlin, Germany

[#] Present address: University of Cambridge, Department of Medicine, Molecular Immunity Unit, MRC Laboratory of Molecular Biology, Cambridge CB2 0QH, UK

* Correspondence to: markus.wahl@fu-berlin.de

## Supplementary Discussion

### Increased stability of a $\rho^{P167L}$-Psu interaction is based on local refolding of the $\rho^{P167L}$ variant

CryoEM/SPA analysis of $\rho^{P167L}$-ATPγS-Psu mixtures yielded two main reconstructions from ~764,000 particle images (Supplementary Table 1; Supplementary Figs. 6-8). The first reconstruction represents two open hexameric $\rho^{P167L}$ rings, in the rotational register observed for $\rho^{wt}$-ATP-Psu complex II (global resolution 3.09 Å; Supplementary Fig. 8). An additional $\rho^{P167L}$ subunit can join at the center of this complex and interlock with the most central $\rho^{P167L}$ subunit of the opposed ring via refolding and domain swapping (complex $II^{locked}$; Fig. 1g; Supplementary Fig. 8). Upon joining of this additional subunit, the helical pitch of the $\rho^{P167L}$(A) ring does not increase, such that the NTD of the additional $\rho^{P167L}$ subunit in its canonical conformation would clash with the most peripheral subunit of the $\rho^{P167L}$(A) ring (Supplementary Fig. 9b). As a consequence, the NTD of the additional $\rho^{P167L}$ subunit is displaced and not defined in the cryoEM reconstruction (cartoon in Supplementary Fig. 9b). The CTD of the additional subunit in its canonical conformation would clash with the CTD of $\rho^{P167L}$(B) subunit 1 (Supplementary Fig. 9b); nevertheless, the additional subunit can be accommodated as both CTDs refold and form domain-swapped β-sheet arrangements (Supplementary Fig. 9b,d). Upon refolding, the Psu interaction interface is altered, preventing Psu association with the central, refolded $\rho^{P167L}$ subunits. Consequently, no additional Psu dimers can join as observed for the higher-oligomeric $\rho^{wt}$-ATP-Psu complex $II^{expanded}$.

In the refolded $\rho^{P167L}$ subunits, the region preceding L167 (residues 141-163), which encompasses the C-terminal part of the hinge region, becomes disordered, as indicated by a lack of interpretable cryoEM density. C-terminally of L167, the $\rho^{wt}$ fold is maintained up to residue L345 (Supplementary Fig. 9d). In the following region up to residue Q378, helical elements are repositioned and changed in register. Between residues Q378 and N401, the helical elements of the canonical fold adopt an extended β-hairpin structure that constitutes the main swapped

element (Supplementary Fig. 9d). The swapped β-hairpins connect the central, six-stranded β-sheets of the CTDs of the domain-swapped $\rho^{P167L}$ subunits into a continuous, 16-stranded sheet (Supplementary Fig. 9d). Residues beyond N401 again lack interpretable cryoEM density.

Mutation-induced domain swapping has been described previously and is frequently associated with destabilization of the fold of the affected protein variants.[1] Indeed, differential scanning fluorimetry revealed that the apparent melting temperature of $\rho^{P167L}$ is decreased by about 7 °C compared to wt ρ (Supplementary Fig. 9e). Thus, the P167L substitution leads to a decrease in the fold stability of ρ subunits that allows refolding of part of the CTD. As a consequence, a more stable interlocking of $\rho^{P167L}$ complexes is possible via the observed domain swapping, leading to the formation of overall more stable $\rho^{P167L}$-Psu complexes.

**P4-like mobile elements encoding Psu are hubs for phage defense genes**

Association of restriction-modification (RM) genes with P4 has been known since the 1980's.[2-4] Recent studies revealed that a large fraction of *E. coli* genomes carries P4-like elements adjacent to anti-phage systems, which included abortive infection (Abi), RM, and other defense modules.[5-7] In P4, Psu is encoded in the *sid-δ-psu* operon and activates expression of *δ* and *psu,* an effect that resembles polarity suppression.[8] This suggests that Psu may also promote expression of associated defense clusters located downstream from the *psu* gene, thus contributing to phage defense.

P4 and P2 can infect diverse bacteria[9]. To explore Psu association with defense modules beyond *Enterobacteriaceae*, we investigated 1,161 bacterial genomes encoding Psu (Supplementary Fig. 13a) and used DefenseFinder[10] to detect putative defense systems in the *psu* neighborhood. This analysis revealed a known defense system in more than a quarter (247) of *psu*$^{+}$ genomes (Supplementary Fig. 13b), inserted between the *psu* and *int* (integrase) genes of P4, adjacent to the *cos* sites (Fig. 9). This location is consistent with frequent recombination and acquisition through horizontal transfer[6]. These defense systems are compact (~3 kb),

presumably reflecting the P4 genome size limitations, and represent 36 known classes (Supplementary Fig. 13c; Supplementary Data 1b,c).

The catalog of known defense systems is far from complete. Bioinformatic screens based on gene syntax identified many novel putative defense gene clusters, which have been shown to encode active defense elements[11-13]. We thus used analogous guilt-by-association criteria to identify 662 putative defense systems in the *psu-int* region that were missed by DefenseFinder (Supplementary Fig. 13b; Supplementary Data 1d) but share the compact size of known defense systems. An HMM search against the Pfam database for the proteins encoded in these loci identified 767 hits from 107 Pfam families that contain nucleases, methylases, toxin-antitoxin and Abi systems, and other components commonly present in phage defense systems (Supplementary Data 1f,g). This analysis suggests that a considerable fraction of the P4 "dark matter" is also involved in transferring defense genes across host genomes, and that new P4-linked defense systems can be found in silico, followed by experimental validation. For example, our analysis identified a sirtuin (Sir2)-HerA helicase module adjacent to Psu in *Klebsiella pneumoniae* (Fig. 9). Although this module was not recognized by DefenseFinder, an analogous Sir2-HerA system from *Paenibacillus* sp. has been recently shown to trigger abortive infection and cell death[14] via the Sir2-mediated depletion of the cellular pool of $NAD^+$, a signature activity of Sir2 in different defense contexts[14-16].

Many defense genes are codirectional with Psu (Fig. 9), potentially extending the *psu* operon and falling under its polarity control[8]. However, many defense loci contain genes that are not colinear, *e.g.*, Psu-linked *Eco*O109I RM system identified by Kita *et al*.[3], or are convergent with the *psu* gene, *e.g.*, a putative defense system shown in Fig. 9. It is important to point out that Psu acts fundamentally differently from canonical phage anti-terminators, which are recruited to RNA polymerase *in cis* and modify the enzyme to become resistant to ρ while transcribing the phage operon.[17] Psu directly binds to ρ, sequestering ρ in an inactive state, and can thus act on any gene silenced by ρ, irrespective of its orientation or location, *in trans.* Consistently, Psu has been

shown to inhibit termination within P2 and P4 operons and at many plasmid and chromosomal loci[8,18-20], but may be expected to exhibit a more potent activity while acting *in cis*. Notably, the defense cassettes identified here are strikingly more AT-rich, ranging from 15 to 25%, than their linked P4-like elements. Therefore, by analogy to other known ρ targets (prophages, EPODs, pathogenicity islands, etc.[18,21]) that have lower GC content than the host genome, we hypothesize that ρ silences these genes while Psu counter-silences ρ.

# Supplementary Tables

## Supplementary Table 1: CryoEM data collection, refinement and validation statistics.

| | **$\rho^{wt}$-ATP-Psu** | | **$\rho^{wt}$-ATPγS-Psu** | | **$\rho^{P167L}$-ATPγS-Psu** | | **$\rho^{P167L}$** |
|---|---|---|---|---|---|---|---|
| | **Complex II** | **Complex $II^{expanded}$** | **Complex III** | **Complex $III^{expanded}$** | **Complex II** | **Complex $II^{locked}$** | **$\rho^{P167L}$-ATPγS** |
| EMDB ID | 51235 | 51236 | 17637 | 17639 | 17640 | 17641 | 51237 |
| PDB ID | 9GCS | 9GCT | 8PEU | 8PEW | 8PEX | 8PEY | 9GCU |
| **Data collection and processing** | | | | | | | |
| Microscope | FEI Titan Krios G3i | | | | | | |
| Voltage [keV] | 300 | | | | | | |
| Camera | Falcon 3EC | | | | | | |
| Magnification | 96,000 | 96,000 | 96,000 | 96,000 | 96,000 | 96,000 | 96,000 |
| Pixel size at detector [Å/pixel] | 0.819 | 0.819 | 0.832 | 0.832 | 0.832 | 0.832 | 0.819 |
| Total electron exposure [$e^{-}/Å^2$] | 44 | 44 | 42 | 42 | 42 | 42 | 44 |
| Exposure rate [$e^{-}$/pixel/s] | 0.7 | 0.7 | 0.7 | 0.7 | 0.7 | 0.7 | 0.7 |
| Frames / exposure | 33 | | | | | | |
| Defocus range [µm] | 0.80 - 2 | | | | | | |
| Automation software | EPU (version 2.8.1) | | | | | | |
| Micrographs collected | 6,452 | 6,452 | 6,066 | 6,066 | 2,766 | 2,766 | 1,473 |
| Micrographs used | 6,409 | 6,409 | 5,986 | 5,986 | 2,723 | 2,723 | 1,470 |
| Total extracted particles | 966,398 | 966,398 | 1,815,462 | 1,815,462 | 823,078 | 823,078 | 804,461 |
| Final particles | 17,296 | 64,053 | 73,056 | 15,407 | 273,935 | 317,635 | 487,613 |
| Point-group or helical symmetry parameters | C1 | C1 | C1 | C1 | C1 | C1 | C1 |
| Global resolution [Å]; $FSC^{a}_{0.143}$ (unmasked / masked) | 7.4 / 3.9 | 4.5 / 3.7 | 4.5 / 3.6 | 7.6 / 4.2 | 3.9 / 3.1 | 3.8 / 2.9 | 3.2 / 2.7 |
| Local resolution range [Å] | 3.0 - 30 | 1.8 – 30.0 | 1.8-30.0 | 3.1-30.0 | 2.2-30.0 | 2.1-30.0 | 2.3 - 30 |
| Map sharpening *B* factor [$Å^2$] / (*B* factor range) | -38 | -70 | -82 | -57 | -81 | -83 | -99 |
| Map sharpening method | Local *B* factor | | | | | | |
| Refinement package | PHENIX (version 1.20_4459); real.space.refine | | | | | | |
| **Model composition** | | | | | | | |
| Non-hydrogen atoms | 54,790 | 73,940 | 57,632 | 83,524 | 54,751 | 55,957 | 20,023 |
| ρ molecules / atoms | 12 / 39,584 | 16 / 52,248 | 12 / 39,588 | 18 / 52,588 | 12 /39,588 | 13 / 41,172 | 6 / 19,800 |
| Psu molecules / atoms | 10 / 14,780 | 14 / 27,320 | 10 / 14,780 | 16 / 23,468 | 10 / 14,780 | 10 / 14,780 | - |
| ATPγS molecules | - | - | 12 | 16 | 12 | 12 | 5 |
| ATP molecules | 12 | 15 | - | - | - | - | - |
| $Mg^{2+}$ ions | 12 | | 12 | 16 | 12 | 12 | 5 |
| Water molecules | - | - | - | - | - | - | 83 |

| Model refinement | | | | | | | |
|---|---|---|---|---|---|---|---|
| **Model-map scores** | | | | | | | |
| CC[b] (mask) | 0.76 | 0.73 | 0.74 | 0.69 | 0.82 | 0.84 | 0.85 |
| CC (volume) | 0.74 | 0.71 | 0.73 | 0.67 | 0.81 | 0.84 | 0.85 |
| **Average *B* factors [Å$^2$]** | | | | | | | |
| Overall | 166 | 184 | 163 | 221 | 162 | 144 | 115 |
| ρ molecules | 169 | 188 | 173 | 237 | 164 | 144 | 267 |
| Psu molecules | 163 | 173 | 156 | 181 | 153 | 145 | - |
| ATPγS molecules | - | - | 202 | 284 | 120 | 122 | 145 |
| ATP moleclules | 195 | 155 | - | - | - | - | - |
| $Mg^{2+}$ ions | 199 | 164 | 221 | 288 | 106 | 110 | 150 |
| Water molecules | - | - | - | - | - | - | 124 |
| **Rmsd[c] from ideal values** | | | | | | | |
| Bond lengths [Å] | 0.002 | 0.003 | 0.002 | 0.003 | 0.003 | 0.003 | 0.003 |
| Bond angles [°] | 0.461 | 0.691 | 0.624 | 0.712 | 0.625 | 0.604 | 0.642 |
| **Validation[d]** | | | | | | | |
| MolProbity score | 1.88 | 1.89 | 1.73 | 1.95 | 1.67 | 1.70 | 1.61 |
| CaBLAM outliers [%] | 1.53 | 1.1 | 0.9 | 1.1 | 1.2 | 1.3 | 1.1 |
| Clashscore | 12.8 | 16.9 | 14.1 | 18.8 | 13.8 | 13.6 | 12.5 |
| Poor rotamers [%] | 2.2 | 0 | 0.0 | 0.0 | 0.0 | 0.0 | 0.1 |
| Cβ deviations | 0 | 0 | 0 | 0 | 0 | 0 | 0 |
| EMRinger score | 0.50 | 0.60 | 0.78 | -0.26 | 1.56 | 1.56 | 2.65 |
| **Ramachandran plot** | | | | | | | |
| Favored [%] | 98.0 | 97.1 | 97.7 | 97.0 | 98.0 | 97.8 | 98.0 |
| Allowed [%] | 2.0 | 2.9 | 2.3 | 3.0 | 2.0 | 2.2 | 2.0 |
| Outliers [%] | 0.0 | 0.0 | 0.0 | 0.0 | 0.0 | 0.0 | 0.0 |
| **Ramachandran *Z*-score (rmsd)** | | | | | | | |
| Whole | 1.04 (0.10) | 0.21 (0.09) | 0.78 (0.10) | -0.07 (0.08) | 0.80 (0.10) | 0.79 (0.10) | 0.35 (0.17) |
| Helix | 1.43 (0.09) | 0.68 (0.07) | 1.15 (0.08) | 0.53 (0.07) | 1.27 (0.09) | 1.33 (0.09) | 0.78 (0.16) |
| Sheet | 0.33 (0.19) | 0.18 (0.17) | 0.32 (0.19) | -0.03 (0.16) | 0.40 (0.20) | 0.05 (0.19) | 0.36 (0.30) |
| Loop | -0.34 (0.13) | -0.58 (0.11) | -0.39 (0.13) | -0.88 (0.10) | -0.46 (0.12) | -0.58 (0.13) | -0.28 (0.18) |

[a] FSC, Fourier shell correlation
[b] CC, correlation coefficient
[c] Rmsd, root-mean-square deviation
[d] Using MolProbity[22]

**Supplementary Table 2: Defective Psu and ρ variants.**

| **Psu variants defective in ρ binding/inhibition** | | |
|---|---|---|
| **Variant** | **Likely effect** | **Reference** |
| L21P | Destabilization of the α1/α2 coiled-coil | [23] |
| V45F | Local mis-folding, disruption of R43 and D49 interactions with Y197 and R146 of ρ | [24,25] |
| S72L | Destabilization of the α1/α2 coiled-coil | [23] |
| E56K | Disruption of the interaction with the backbone amide of ρ Q374 | [23] |
| Δ95-98 | Disruption of Psu dimerization | [19] |
| Δ97-100 | Disruption of Psu dimerization | |
| P157L | Destabilization of Psu dimer | [23] |
| P157S | Destabilization of Psu dimer | [23] |
| R166C | Effect on helix α7 positioning | [23] |
| R166P | Effect on helix α7 positioning | [23] |
| F169V | Effect on helix α7 positioning through stacking with W60 | [19] |
| ΔCTD10 | Partial deletion of helix α7 interaction interface | [26] |
| ΔCTD20 | Deletion of helix α7 interaction interface | [26] |
| **ρ variants defective in Psu binding/resistant to Psu** | | |
| **Variant** | **Likely effect** | **Reference** |
| R144E | Local mis-folding, effect on interaction of R146 with D49 of Psu | [27] |
| Δ144-148 | Local mis-folding, disruption of interaction with D49 of Psu | [27] |
| R146E | Disruption of interaction with D49 of Psu | [27] |
| E148R | Local mis-folding | [27] |
| R149E | Disruption of stacking with Y197 of ρ to position it for interaction with R43 of Psu | [27] |
| Δ149-153 | Local mis-folding, effect on the positioning of Y197 of ρ for interaction with R43 of Psu | [27] |
| N151D | Effect on the positioning of Y197 of ρ for interaction with R43 of Psu | [27] |

**Supplementary Table 3: PCR primers (5' to 3').**

| Primer | Sequence | Usage |
|---|---|---|
| DG30 | TCAGGAGAAGGCGCAAACTGCAGTCGC | FP[a] to generate *psu*-R43A in pET21b |
| DG31 | GCGACTGACGTTTGCGCCTTCTCCTGA | RP[b] to generate *psu*-R43A in pET21b |
| DG32 | GTCAGTCGCCTGCGCGAATTACGCAAT | FP to generate *psu*-D49R in pET21b |
| DG33 | ATTGCGTAATTCGCGCAGGCGACTGAC | RP to generate *psu*-D49R in pET21b |
| RS84new | GCG CGCGCCATATGAATCTTACCGAATTAAAG | FP upstream of *rho* with *Nde*I site to generate *rho*-Y197A in pET21b |
| RS85 | GCGCGCCTCGAGTTATGAGCGTTTCATCATTTC | RP downstream of *rho* with *Xho*I site and with stop codon to generate *rho*-Y197A in pET21b |
| RS123 | GCGCGCGCCATATGGAAAGCACAGCCTTACAGCAGGCC | FP upstream of *psu* with *Nde*I site to generate *psu*-D49R, *psu*-R43A and *psu*-K180A in pET28b |
| RS124 | GCGCGCCTCGAGCACTGACTGACGTGATGCCAGTTGC | RP downstream of *psu* with *Xho*I and without stop codon to generate *psu*-D49R, *psu*-R43A and *psu*-K180A in pET28b |
| RS2178 | CAGAAACGTCAGTCGCCTGCGCGAATTACGCAATATTATCG | FP to generate *psu*-D49R by SDM[c] |
| RS2179 | CGATAATATTGCGTAATTCGCGCAGGCGACTGACGTTTCTG | RP to generate *psu*-D49R by SDM |
| RS2180 | TCTGTCAGGAGAAGGCGCAAACGTCAGTCGCCTG | FP to generate *psu*-R43A by SDM |
| RS2181 | CAGGCGACTGACGTTTGCGCCTTCTCCTGACAGA | RP to generate *psu*-R43A by SDM |
| RS2182 | AGAACATGATTTTTTCGCGTGCAAGTGCGCAACTGGCATCAC | FP to generate *psu*-K180A by SDM |
| RS2183 | GTGATGCCAGTTGCGCACTTGCACGCGAAAAAATCATGTTCT | RP to generate *psu*-K180A by SDM |
| RS2224 | GAGCATTGCTGCCAACCACCCGG | FP to generate *rho*-Y197A by SDM |
| RS2225 | TGAGCAATGTTCTGCAGC | RP to generate *rho*-Y197A by SDM y |

[a] FP, forward primer
[b] RP, reverse primer
[c] SDM, site-directed mutagenesis

**Supplementary Table 4: Plasmids and strains.**

| Plasmid | Description | Source |
|---|---|---|
| pETM11-*rho* | pETM11 with wt *rho*; no encoded tag; *Kan*$^{R}$ | [28] |
| pUC18-λ$t_{R1}$-*rut* | pUC19 with the λ$t_{R1}$ *rut* region cloned at *Xba*I/*Kpn*I sites; *Amp*$^{R}$ | [29] |
| pRS455 | pET21b with wt *psu* cloned at *Nde*I/*Xho*I sites; no encoded tag; *Amp*$^{R}$ | This study |
| pRS964 | pET21b with *rho*-P167L cloned at *Nde*I/*Xho*I sites; no encoded tag; *Amp*$^{R}$ | [27] |
| pRS100 | pET21b with wt *rho* cloned at *Nde*I/*Xho*I sites; no encoded tag; *Amp*$^{R}$ | [26] |
| pRS259 | pNL150 with *psu*-F169V; *Cam*$^{R}$ | [19] |
| pRS458 | pET28b with wt *psu* cloned at *Nde*I/*Xho*I sites; encoded N-terminal His-tag; *Kan*$^{R}$ | [26] |
| pRS555 | pNL150 with *psu*-E56K; *Cam*$^{R}$ | [26] |
| pRS624 | pET28b with *psu*-E56K cloned at *Nde*I/*Xho*I sites; encoded N-terminal His-tag; *Kan*$^{R}$ | [23] |
| pRS649 | pCL1920 with wt *rho* with its promoter cloned at *Hind*III/*Sac*I sites; *Spec*$^{R}$, *Strep*$^{R}$ | [30] |
| pRS1117 | pNL150 with wt *psu* | [19] |
| pRS1199 | RS649 with *rho*-R146E; *Spec*$^{R}$, *Strep*$^{R}$ | [27] |
| pRS1219 | pET21b with *rho*-R146E cloned at *Nde*I/*Xho*I site; no encoded tag; *Amp*$^{R}$ | [27] |
| pRS2158 | pNL150 with *psu*-D49R; *Cam*$^{R}$ | This study |
| pRS2159 | pNL150 with *psu*-K180A; *Cam*$^{R}$ | This study |
| pRS2160 | pNL150 with *psu*-R43A; *Cam*$^{R}$ | This study |
| pRS2194 | RS649 with *rho*-Y197A; *Spec*$^{R}$, *Strep*$^{R}$ | This study |
| pRS2236 | pET21b with Y197A *rho* cloned at *Nde*I/*Xho*I site, non-His-tag; *Amp*$^{R}$ | This study |
| pRS2237 | pET28b with R43A *psu* cloned at *Nde*I/*Xho*I site, His-tag at N-terminal; *Kan*$^{R}$ | This study |
| pRS2238 | pET28b with D49R *psu* cloned at *Nde*I/*Xho*I site, His-tag at N-terminal; *Kan*$^{R}$ | This study |
| pRS2239 | pET28b with K180A *psu* cloned at *Nde*I/*Xho*I site, His-tag at N-terminal; *Kan*$^{R}$ | This study |

| Strain | Genotype | Source |
|---|---|---|
| RS1263 | *E. coli* MG1655; K-12 | Lab stock |
| RS1309 | *E. coli* MG1655*ΔrhoΔrac*, with shelter plasmid pHYD1201 (*Amp*$^{R}$) | [31] |
| RS2047 | MG1655*ΔracΔlac*, λRS45 lysogen carrying $P_{lac}$-λ$t_{R1}$-*lacZYA* | [32] |

## Supplementary Figures

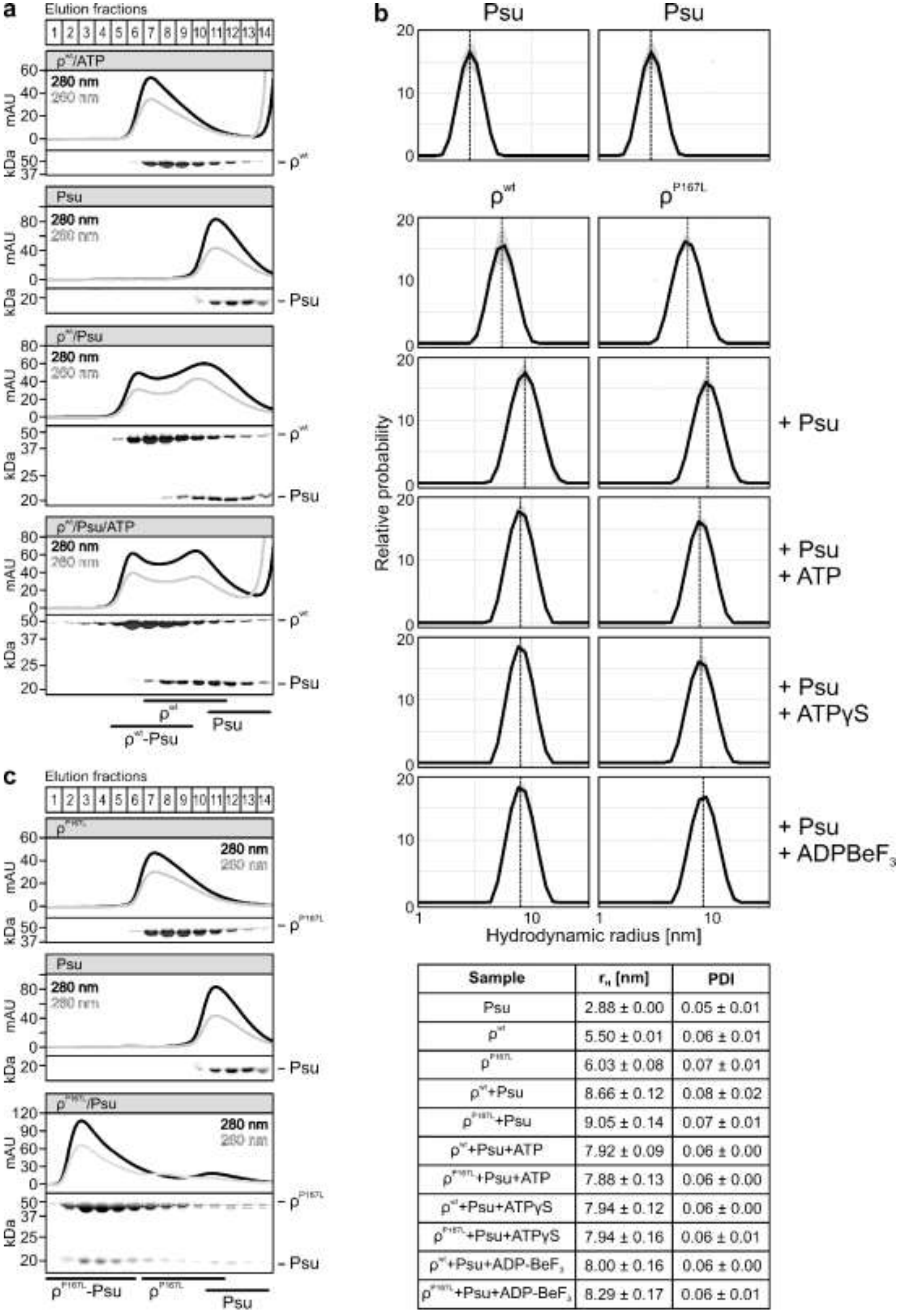


| Sample | $r_H$ [nm] | PDI |
|---|---|---|
| Psu | 2.88 ± 0.00 | 0.05 ± 0.01 |
| $\rho^{wt}$ | 5.50 ± 0.01 | 0.06 ± 0.01 |
| $\rho^{P167L}$ | 6.03 ± 0.08 | 0.07 ± 0.01 |
| $\rho^{wt}$+Psu | 8.66 ± 0.12 | 0.08 ± 0.02 |
| $\rho^{P167L}$+Psu | 9.05 ± 0.14 | 0.07 ± 0.01 |
| $\rho^{wt}$+Psu+ATP | 7.92 ± 0.09 | 0.06 ± 0.00 |
| $\rho^{P167L}$+Psu+ATP | 7.88 ± 0.13 | 0.06 ± 0.00 |
| $\rho^{wt}$+Psu+ATPγS | 7.94 ± 0.12 | 0.06 ± 0.00 |
| $\rho^{P167L}$+Psu+ATPγS | 7.94 ± 0.16 | 0.06 ± 0.01 |
| $\rho^{wt}$+Psu+ADP-$BeF_3$ | 8.00 ± 0.16 | 0.06 ± 0.00 |
| $\rho^{P167L}$+Psu+ADP-$BeF_3$ | 8.29 ± 0.17 | 0.06 ± 0.01 |

**Supplementary Fig. 1: ρ-Psu interaction in solution.**

**a,c**, 260 and 280 nm absorbance traces (top) and SDS-PAGE analyses (bottom) of analytical SEC elution runs monitoring the interaction of $\rho^{wt}$ (**a**) or $\rho^{P167L}$ (**c**) with Psu. Elution fractions are indicated at the top. The same elution fractions were analyzed for each run and were aligned below each other. Proteins and protein mixtures analyzed are indicated above each gel. Molecular mass markers are indicated on the left. Protein bands are identified on the right. Fractions containing isolated proteins or complexes are identified below each gel. Experiments were repeated independently at least two times with similar results.

**b**, Top, DLS measurements to determine particle sizes of Psu, ρ, $\rho^{P167L}$ and indicated mixtures. Graphs show the relative probability of detected particle sizes against the hydrodynamic radius calculated from intensity-weighted size distribution fits. Dashed vertical lines, average hydrodynamic radius determined from cumulant analyses across all acquisitions. Gray opaque areas, standard deviation of merged triplicates. Bottom, hydrodynamic radii ($r_H$) and polydispersity indices (PDI) of the tested samples. The PDI describes the presence and spread of different size particle populations in a sample. PDI ≤ 0.1, high monodispersity; 0.1 < PDI ≤ 0.3, predominantly monodisperse samples; 0.3 < PDI ≤ 0.7, polydispersity.

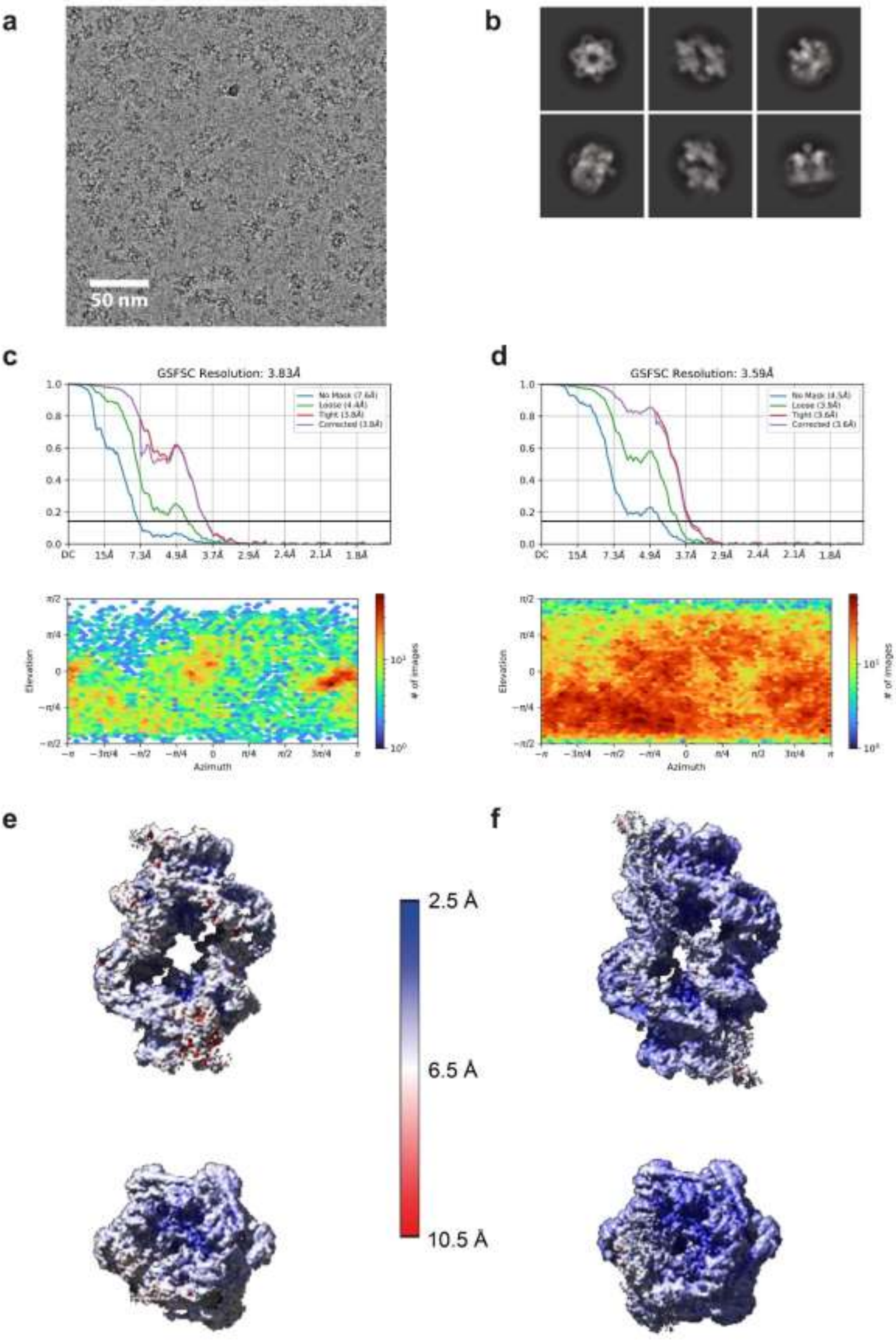
a
b
c
d
e
f
50 nm
GSFSC Resolution: 3.83Å
GSFSC Resolution: 3.59Å
Elevation
Azimuth
# of images
2.5 Å
6.5 Å
10.5 Å

**Supplementary Fig. 2: CryoEM/SPA analysis of a ρ-ATP-Psu complexes.**

**a**, Representative cryoEM micrograph of the ρ-ATP-Psu complexes. Scale bar, 50 nm. Particle images were extracted from 6,409 high-quality micrographs.

**b**, Representative 2D class averages of ρ-ATP-Psu particle images after reference-free 2D classification.

**c,d**, Upper panels, global resolution estimation for cryoEM reconstructions of ρ-ATP-Psu complex II (**c**) and complex $II^{expanded}$ (**d**) by gold-standard Fourier shell correlation (FSC). Black line, $FSC_{0.143}$. Lower panel, viewing direction distribution plots of the cryoEM reconstructions of ρ-ATP-Psu complex II (**c**) and complex $II^{expanded}$ (**d**) as obtained during non-uniform (NU) refinement with cryoSPARC.

**e,f**, Local resolution estimation as determined with cryoSPARC, ranging from 2.5 Å to 10.5 Å for cryoEM reconstructions of ρ-ATP-Psu complex II (**e**) and complex $II^{expanded}$ (**f**).

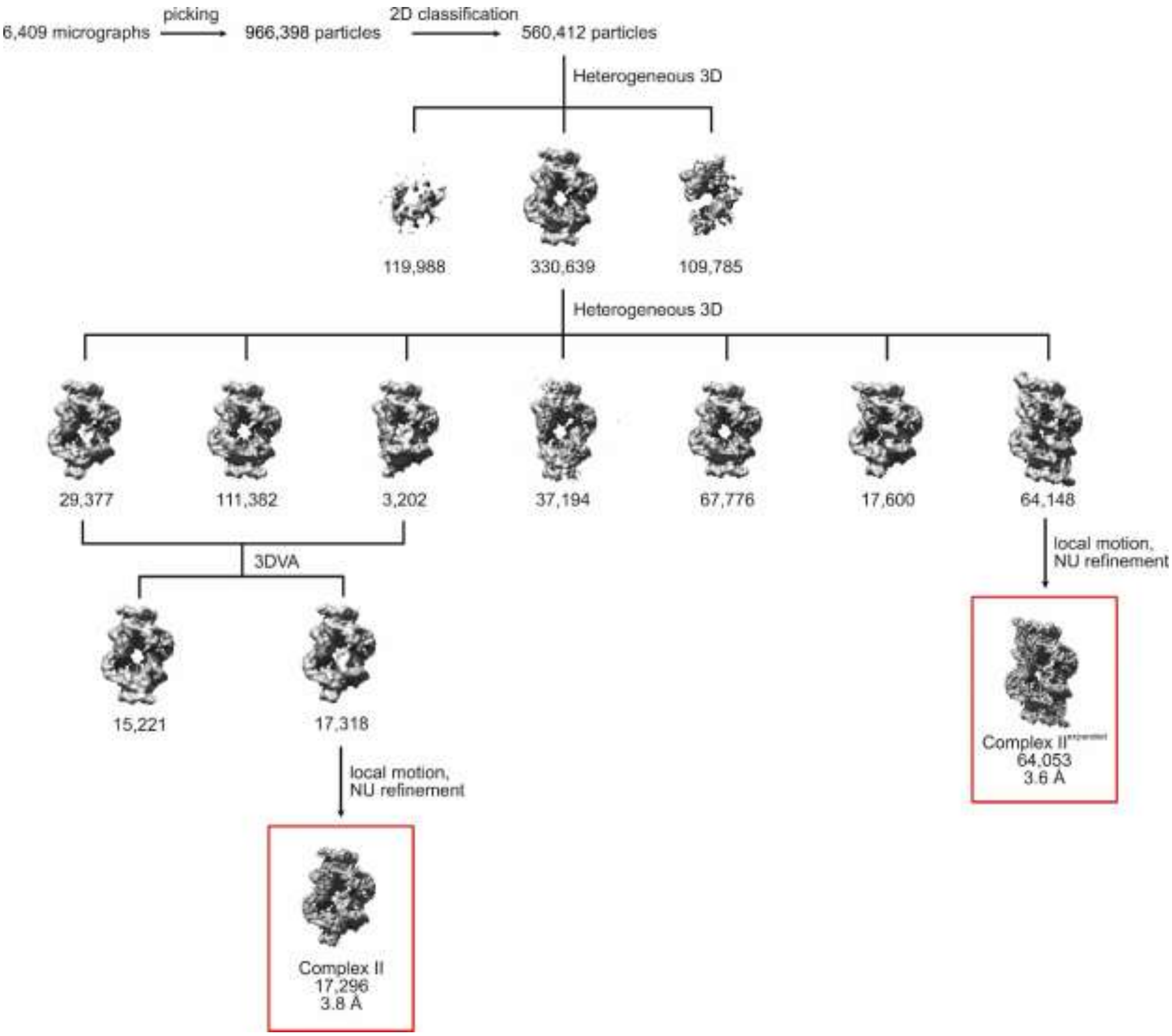


**Supplementary Fig. 3: ρ-ATP-Psu cryoEM data refinement.**

966,398 particles were picked from 6,409 micrographs and subjected to reference-free 2D classification. 560,412 particle images were selected for heterogeneous 3D refinement into 3 classes. The best-appearing class, consisting of 330,639 particle images, was subjected to heterogeneous 3D refinement generating seven classes. Two of these were combined (left branch) and further classified by 3DVA into two classes. Local motion correction and NU refinement yielded a reconstruction at 3.8 Å resolution (complex II). The third cluster (right branch)

was further refined after local motion correction by NU refinement, yielding a reconstruction at 3.6 Å resolution (complex $II^{expanded}$).

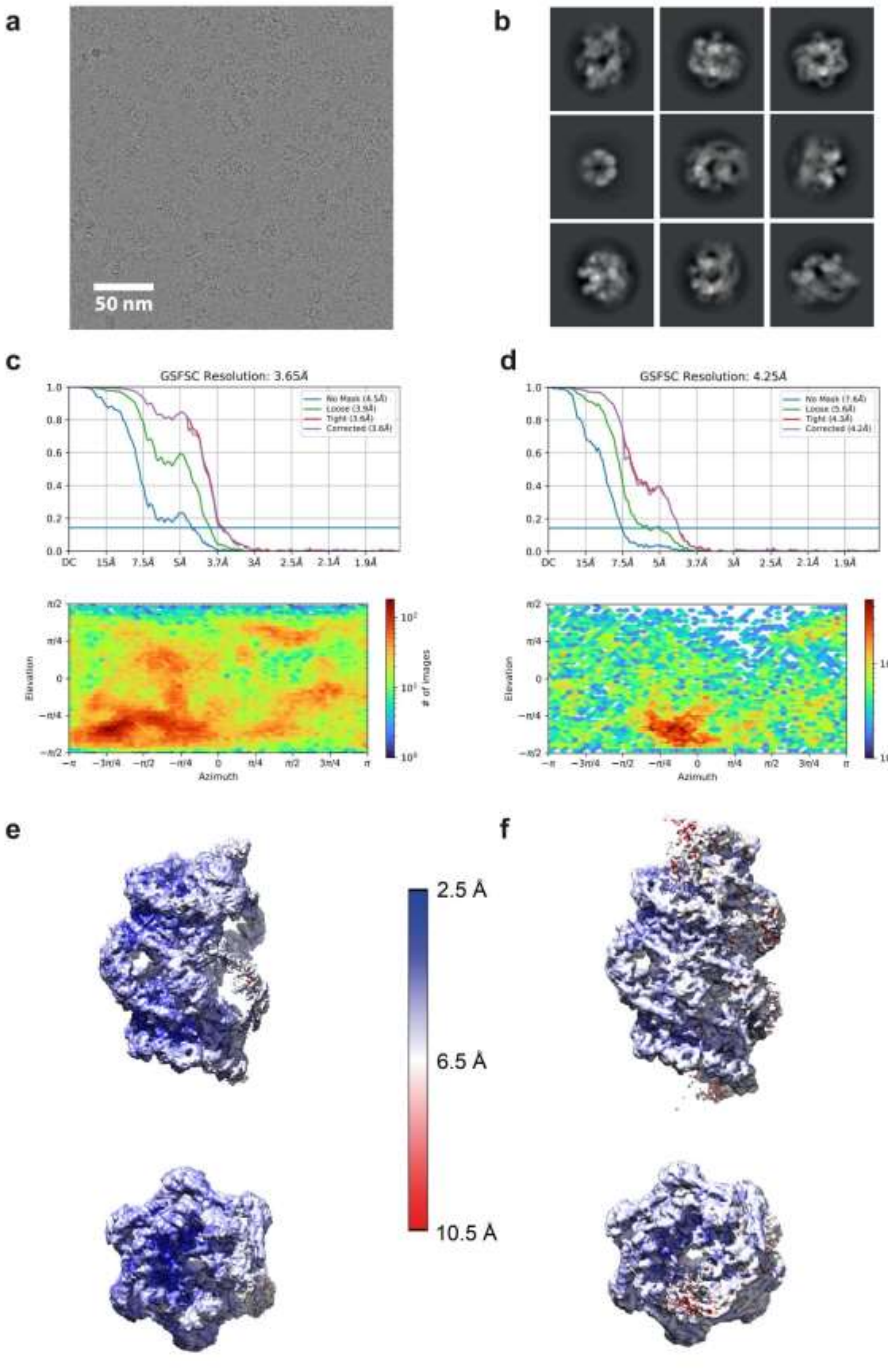

a
50 nm
b
c
GSFSC Resolution: 3.65Å
No Mask (4.5Å)
Loose (3.9Å)
Tight (3.6Å)
Corrected (3.6Å)
DC
15Å
7.5Å
5Å
3.7Å
3Å
2.5Å
2.1Å
1.9Å
Elevation
Azimuth
# of images
d
GSFSC Resolution: 4.25Å
No Mask (7.6Å)
Loose (5.6Å)
Tight (4.3Å)
Corrected (4.2Å)
e
f
2.5 Å
6.5 Å
10.5 Å

**Supplementary Fig. 4: CryoEM/SPA analysis of a ρ-ATPγS-Psu complexes.**

**a**, Representative cryoEM micrograph of the ρ-ATPγS-Psu complexes. Scale bar, 50 nm. Particles were picked from 5,986 high-quality micrographs.

**b**, Representative 2D class averages of ρ-ATPγS-Psu particle images after reference-free 2D classification.

**c,d**, Upper panels, global resolution estimation for cryoEM reconstructions of ρ-ATPγS-Psu complex III (**c**) and complex $III^{expanded}$ (**d**) by gold-standard Fourier shell correlation (FSC). Blue line, $FSC_{0.143}$. Lower panels, viewing direction distribution plots of the cryoEM reconstructions of ρ-ATPγS-Psu complex III (**c**) and complex $III^{expanded}$ (**d**) as obtained during NU refinement with cryoSPARC.

**e,f**, Local resolution estimation as determined with cryoSPARC, ranging from 2.5 Å to 10.5 Å for cryoEM reconstructions of ρ-ATP-Psu complex III (**e**) and complex $III^{expanded}$ (**f**).

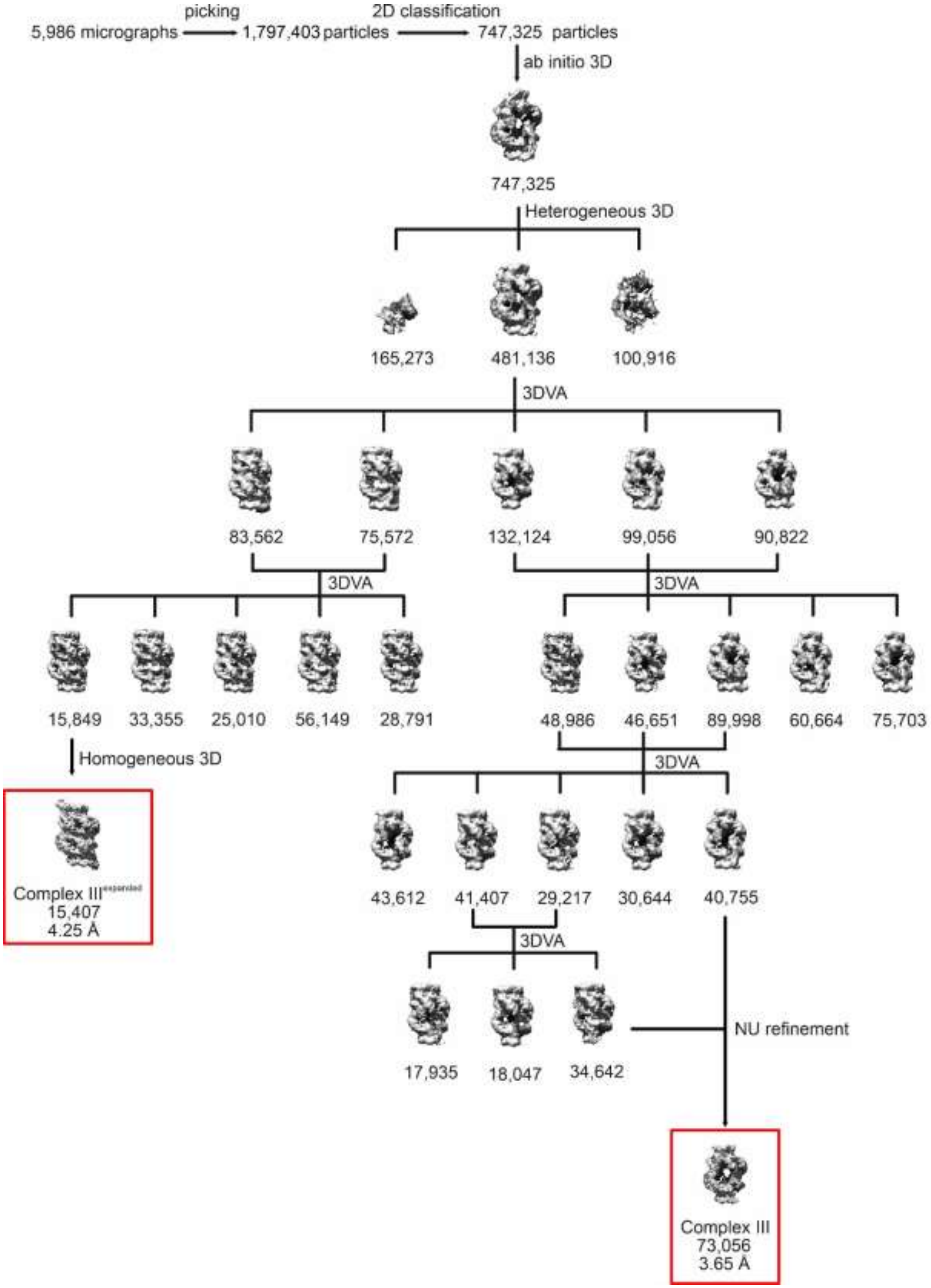

picking
2D classification
5,986 micrographs
1,797,403 particles
747,325 particles
ab initio 3D
747,325
Heterogeneous 3D
165,273
481,136
100,916
3DVA
83,562
75,572
132,124
99,056
90,822
3DVA
3DVA
15,849
33,355
25,010
56,149
28,791
48,986
46,651
89,998
60,664
75,703
Homogeneous 3D
3DVA
Complex III
15,407
4.25 Å
43,612
41,407
29,217
30,644
40,755
3DVA
17,935
18,047
34,642
NU refinement
Complex III
73,056
3.65 Å

**Supplementary Fig. 5: ρ-ATPγS-Psu cryoEM data refinement.**

1,815,462 particle images were picked from 5,986 micrographs and subjected to reference-free 2D classification. 747,325 particle images were selected for iterative cycles of heterogeneous 3D refinement into 3 classes. The best-appearing class, consisting of 481,136 particle images, was selected and subjected to 3D variability analysis (3DVA) generating five classes. Two of these were combined (left branch) and further classified by subsequent 3DVA into five classes, one of which, consisting of 15,849 particles was subjected to NU 3D refinement, which yielded a reconstruction at 4.25 Å resolution (complex $III^{expanded}$). The residual three classes (right branch) were further classified by iterative 3DVA cycles. Two of the resulting classes appeared virtually identical and were combined for NU refinement yielding a reconstruction at 3.65 Å resolution (complex III).

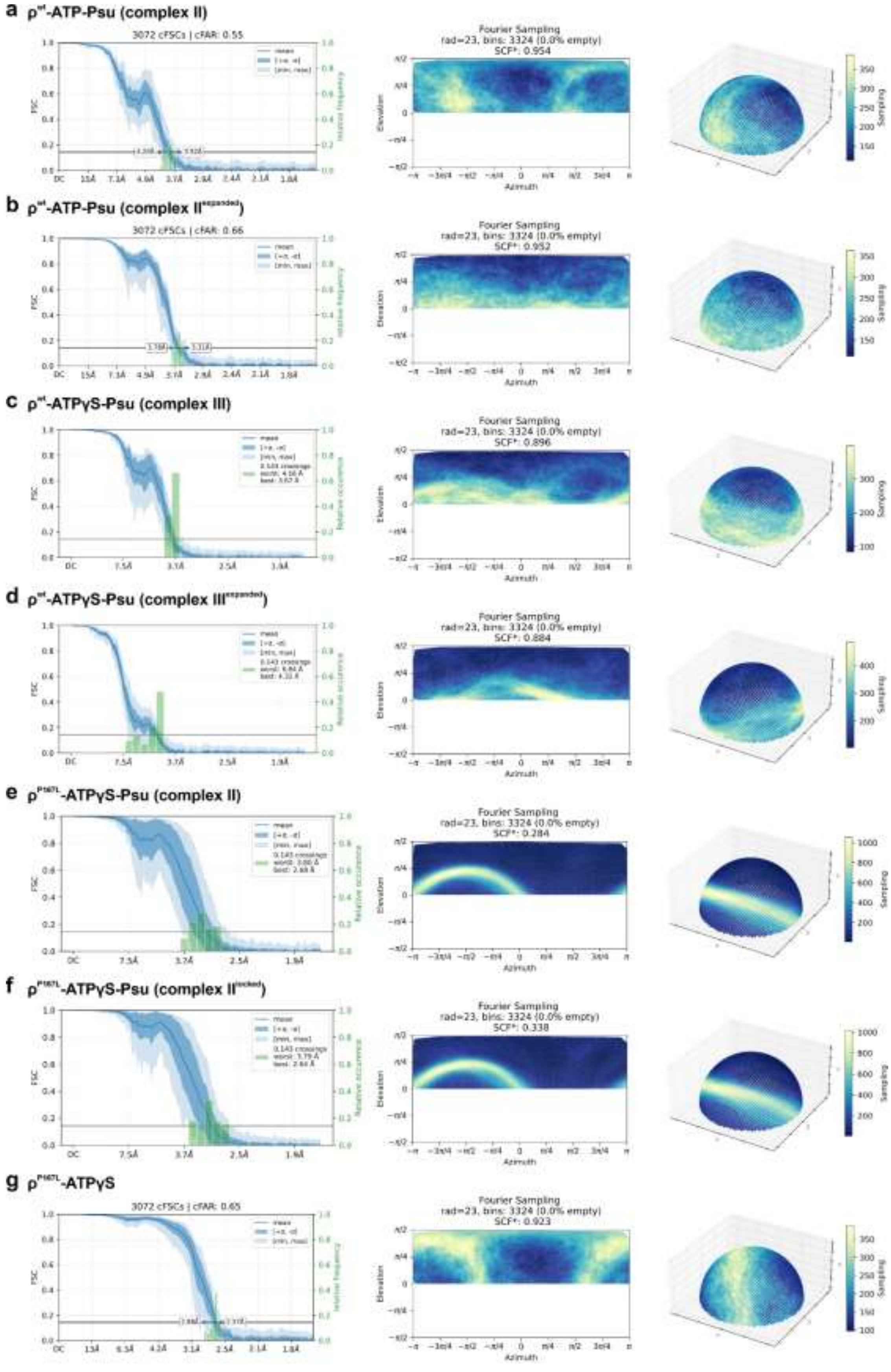
a ρwt-ATP-Psu (complex II)
3072 cFSCs | cFAR: 0.55
Fourier Sampling
rad=23, bins: 3324 (0.0% empty)
SCF*: 0.954
b ρwt-ATP-Psu (complex IIexpanded)
3072 cFSCs | cFAR: 0.66
Fourier Sampling
rad=23, bins: 3324 (0.0% empty)
SCF*: 0.952
c ρwt-ATPγS-Psu (complex III)
Fourier Sampling
rad=23, bins: 3324 (0.0% empty)
SCF*: 0.896
d ρwt-ATPγS-Psu (complex IIIexpanded)
Fourier Sampling
rad=23, bins: 3324 (0.0% empty)
SCF*: 0.884
e ρP167L-ATPγS-Psu (complex II)
Fourier Sampling
rad=23, bins: 3324 (0.0% empty)
SCF*: 0.284
f ρP167L-ATPγS-Psu (complex IIlocked)
Fourier Sampling
rad=23, bins: 3324 (0.0% empty)
SCF*: 0.338
g ρP167L-ATPγS
3072 cFSCs | cFAR: 0.65
Fourier Sampling
rad=23, bins: 3324 (0.0% empty)
SCF*: 0.923
FSC
Elevation
Azimuth
Sampling

**Supplementary Fig. 6: Resolution anisotropy analysis of in vitro reconstituted ρ-Psu complexes.**

**a-g**, Analysis of orientation bias for the cryoEM reconstructions reported in this study. Orientation bias was determined by conical Fourier shell correlation (cFSC) analysis[33] and sampling compensation factor (SCF) analysis[34] as implemented in cryoSPARC. Samples generally showed very little orientation bias with cFSC values greater than 0.5 and SCF above 0.8. Resolution anisotropy is only observed for reconstructions of $\rho^{P167L}$-ATPγS-Psu complexes.

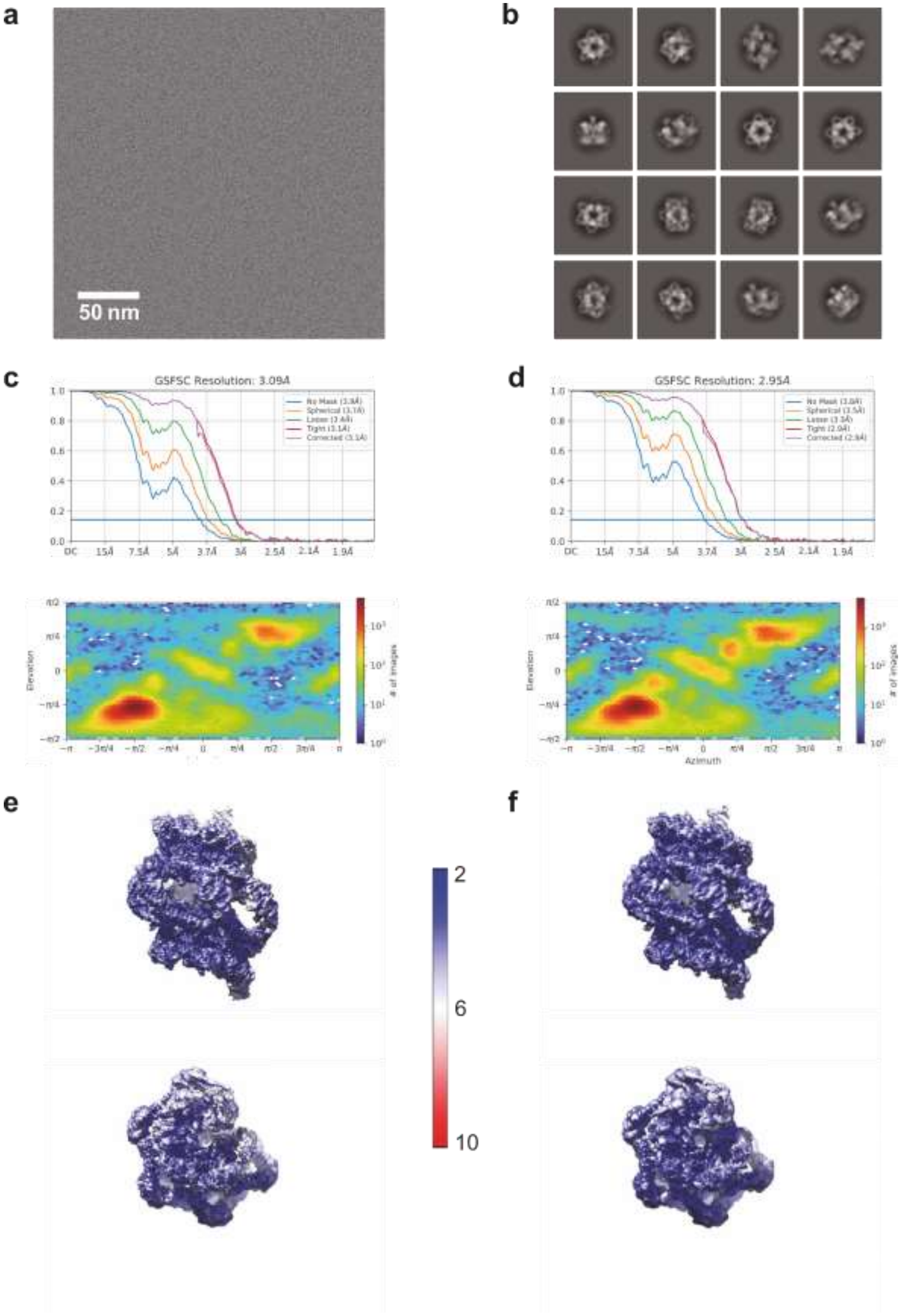
a
50 nm
b
c
GSFSC Resolution: 3.09Å
d
GSFSC Resolution: 2.95Å
Elevation
Azimuth
# of images
e
f
2
6
10

**Supplementary Fig. 7: CryoEM/SPA analysis of $\rho^{P167L}$-ATPγS-Psu complexes.**

**a**, Representative cryoEM micrograph of the $\rho^{P167L}$-ATPγS-Psu complexes. Scale bar, 50 nm. 2,766 micrographs were imaged, particles were picked from 2,723 high-quality micrographs.

**b**, Representative 2D class averages of $\rho^{P167L}$-ATPγS-Psu particle images after reference-free 2D classification.

**c,d**, Upper panels, global resolution estimation for cryoEM reconstructions of $\rho^{P167L}$-ATPγS-Psu complex II (**c**) and complex $II^{locked}$ (**d**) by gold-standard Fourier shell correlation (FSC). Blue line, $FSC_{0.143}$. Lower panels, viewing direction distribution plots of the cryoEM reconstructions of $\rho^{P167L}$-ATPγS-Psu complex II (**c**) and complex $II^{locked}$ (**d**) as obtained during NU refinement with cryoSPARC.

**e,f**, Local resolution estimation as determined with cryoSPARC, ranging from 2 Å to 10 Å for cryoEM reconstructions of $\rho^{P167L}$-ATPγS-Psu complex II (**e**) and complex $II^{locked}$ (**f**).

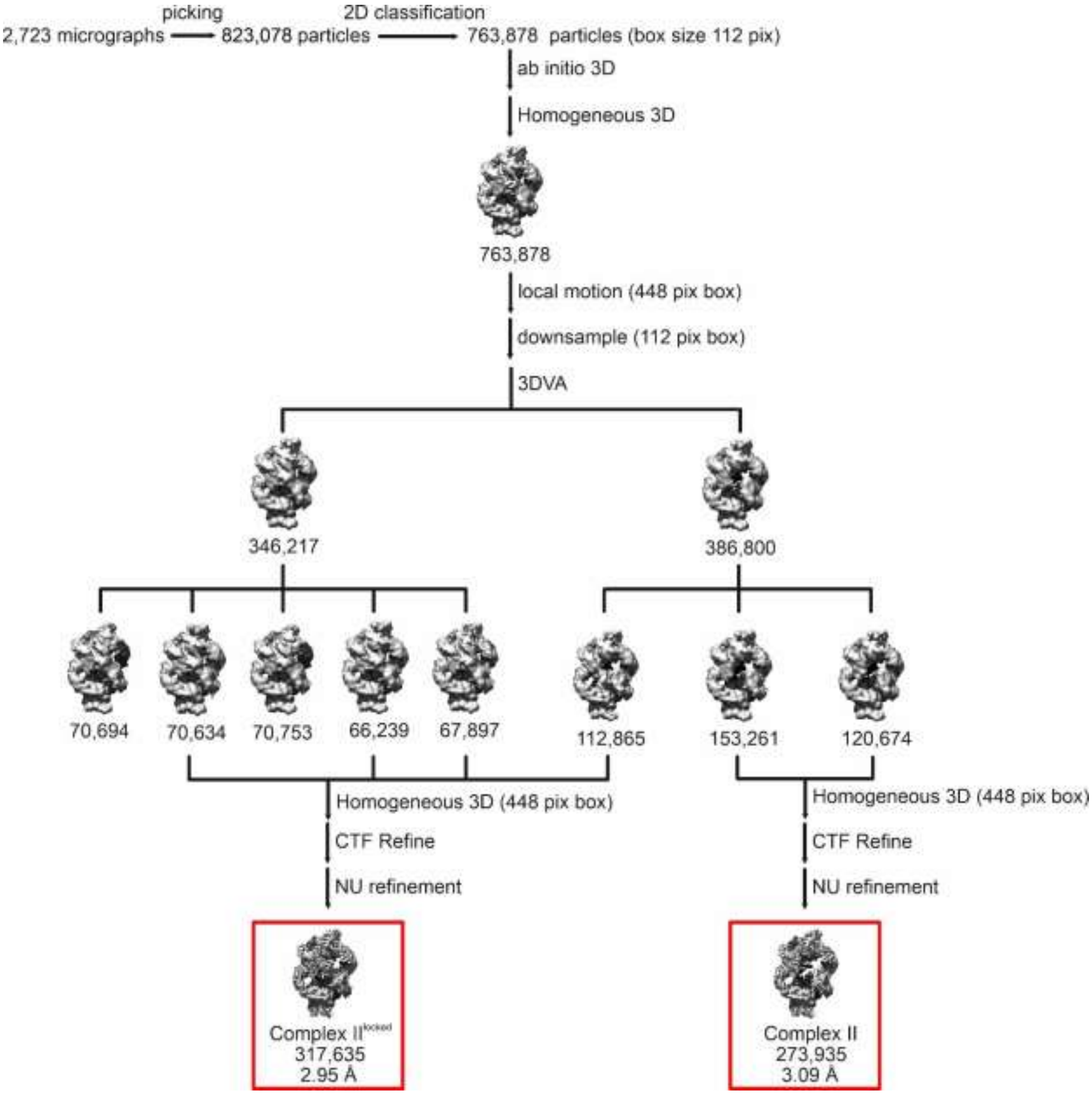


**Supplementary Fig. 8: ρ$^{P167L}$-ATPγS-Psu cryoEM data refinement.**

823,078 particles were picked from 2,723 micrographs and subjected to reference-free 2D classification. 763,878 particle images were selected and used for ab initio 3D reconstruction, followed by homogeneous 3D refinement. Particles were subjected to local motion correction and re-extracted with a box size of 448 px. After down-sampling to a box size of 112 px, 3DVA was applied to separate the dataset into two classes. Each of these was individually subjected to

3DVA, yielding five and three classes, respectively. Similar appearing classes of both branches were combined and homogeneously refined followed by global and local CTF refinement. Final NU refinement yielded reconstructions of 273,935 particle images at 3.09 Å resolution (complex II) and 317,635 particle images at 2.95 Å resolution (complex $II^{locked}$).

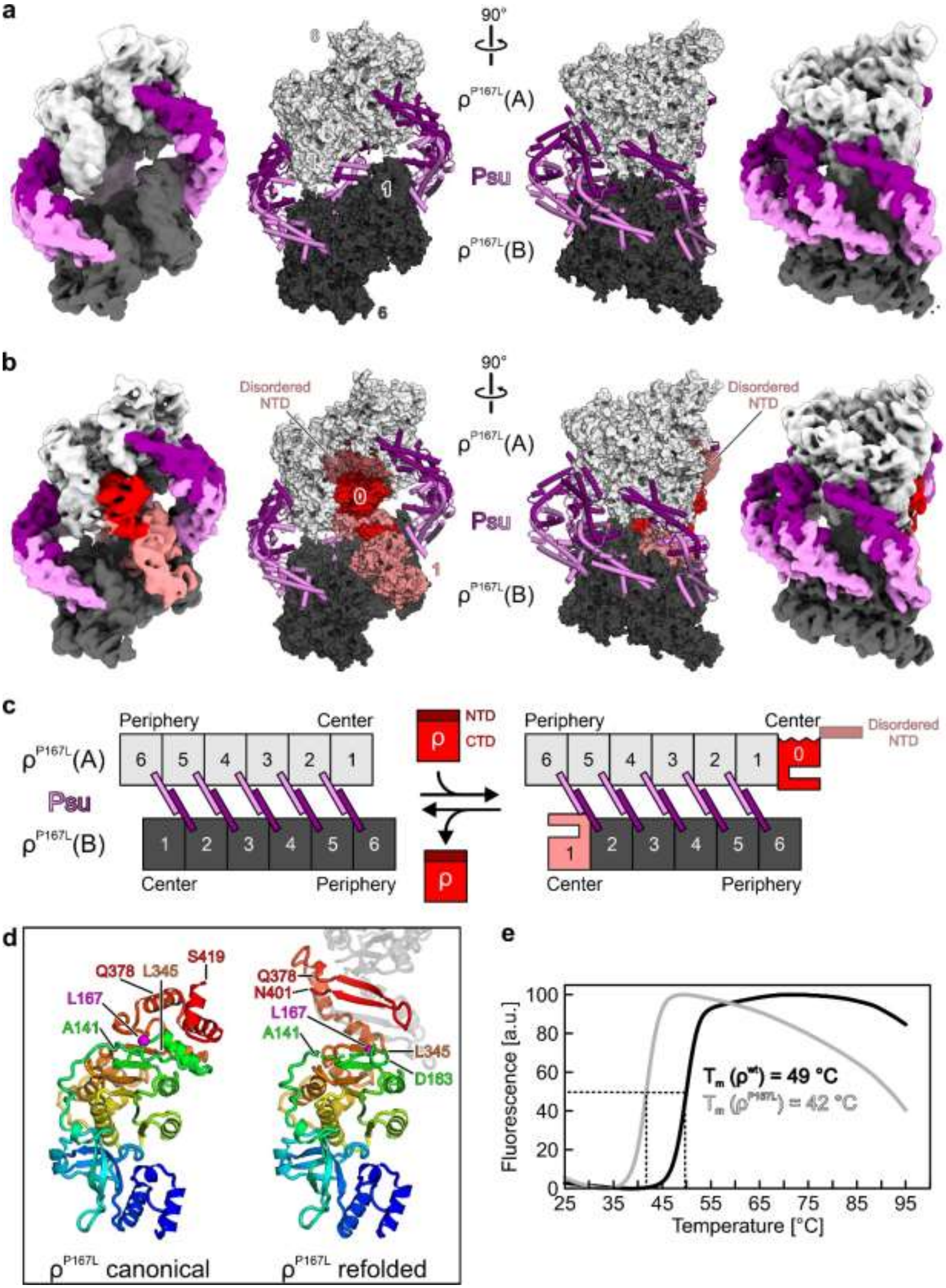
a
90°
ρ$^{P167L}$(A)
Psu
ρ$^{P167L}$(B)
b
Disordered NTD
90°
ρ$^{P167L}$(A)
Psu
ρ$^{P167L}$(B)
c
Periphery
Center
ρ$^{P167L}$(A)
Psu
ρ$^{P167L}$(B)
NTD
CTD
Disordered NTD
d
Q378
L345
S419
L167
A141
N401
D183
ρ$^{P167L}$ canonical
ρ$^{P167L}$ refolded
e
Fluorescence [a.u.]
Temperature [°C]
$T_m$ ($\rho^{wt}$) = 49 °C
$T_m$ ($\rho^{P167L}$) = 42 °C

**Supplementary Fig. 9: Structures of $\rho^{P167L}$-ATPγS-Psu complexes.**

**a**, Orthogonal views of the cryoEM reconstruction (left and right; 6 Å resolution, low-pass-filtered) and model (center) of $\rho^{P167L}$-ATPγS-Psu complex II. Five Psu dimers bridge two open $\rho^{P167L}$ hexamers, ρ(A) and ρ(B). $\rho^{P167L}$ subunits are designated by increasing Arabic numerals from the centers to the peripheries of the complex. Coloring as in Fig. 1.

**b**, Orthogonal views of the cryoEM reconstruction (left and right; 6 Å resolution, low-pass-filtered) and model (center) of $\rho^{P167L}$-ATPγS-Psu complex $II^{locked}$. Five Psu dimers bridge an open $\rho^{P167L}$ heptamer, $\rho^{P167L}$(A), with an open $\rho^{P167L}$ hexamer $\rho^{P167L}$(B). Portions of $\rho^{P167L}$(A) and $\rho^{P167L}$(B) equivalent to $\rho^{P167L}$-ATPγS-Psu complex II are shown in the same colors as in (**a**). Additional $\rho^{P167L}$ subunit 0 in $\rho^{P167L}$(A), red (refolded CTD). Refolded subunit 1 of $\rho^{P167L}$(B), light red. The N-terminal domain modeled in canonical conformation onto the additional $\rho^{P167L}$(A) subunit 0 (semitransparent dark red surface in the models) would clash with $\rho^{P167L}$(A) subunit 6; it is, therefore, displaced and not defined in the cryoEM reconstruction.

**c**, Schemes illustrating $\rho^{P167L}$-Psu interaction patterns in $\rho^{P167L}$-ATPγS-Psu complexes II and $II^{locked}$ and how $\rho^{P167L}$-ATPγS-Psu complexes $II^{locked}$ could emerge from complexes II.

**d**, Side-by-side comparison of a $\rho^{P167L}$ subunit in canonical conformation (left) and in the refolded conformation (right). The models are colored blue to red from N- to C-terminus. For the refolded model, a second refolded $\rho^{P167L}$ subunit, interacting via domain-swap in $\rho^{P167L}$-ATPγS-Psu complex II, is shown as a gray, semi-transparent cartoon. Cα atom of L167, magenta sphere. Positions of landmark residues used to describe the rearrangement are indicated.

**e**, Differential scanning fluorimetry monitoring fold stabilities of wt ρ (black curve) and $\rho^{P167L}$ (gray curve). $T_m$ values were determined as the maxima of the first derivatives of the melting curves. Experiments were repeated independently at least three times with similar results.

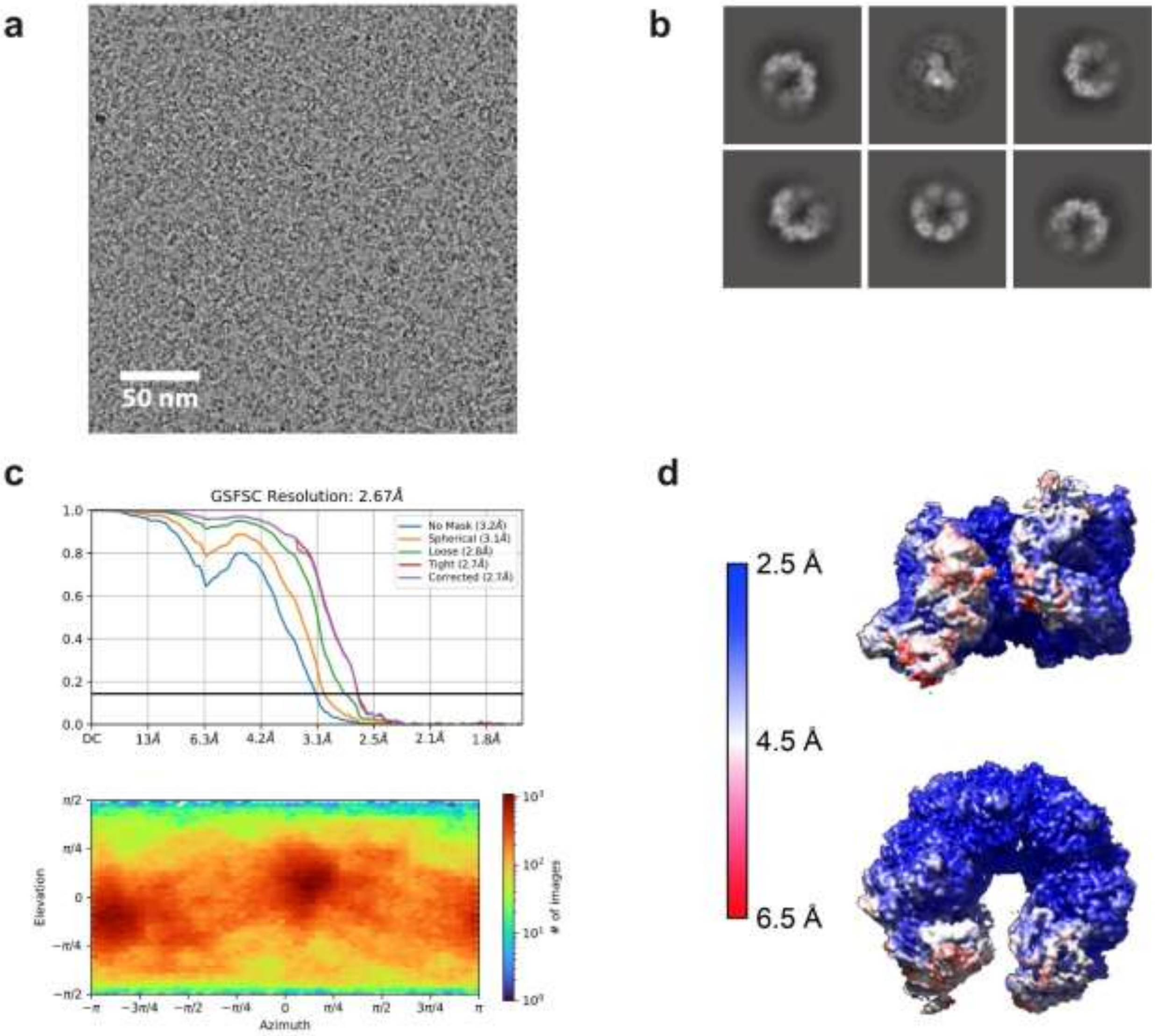


**Supplementary Fig. 10: CryoEM/SPA analysis of $\rho^{P167L}$-ATPγS complexes.**

**a**, Representative cryoEM micrograph of the $\rho^{P167L}$-ATPγS complexes. Scale bar, 50 nm. Particles were picked from 1,470 high-quality micrographs.

**b**, Representative 2D class averages of $\rho^{P167L}$-ATPγS particle images after reference-free 2D classification.

**c**, Upper panel, global resolution estimation for cryoEM reconstructions of a $\rho^{P167L}$-ATPγS complex by gold-standard Fourier shell correlation (FSC). Black line, $FSC_{0.143}$. Lower panel,

viewing direction distribution plot of the cryoEM reconstruction of a $\rho^{P167L}$-ATPγS complex as obtained during NU refinement with cryoSPARC.

**d**, Local resolution estimation as determined with cryoSPARC, ranging from 2.5 Å to 6.5 Å for the cryoEM reconstruction of a $\rho^{P167L}$-ATPγS complex. Despite the addition of ATPγS during sample preparation, the cryoEM reconstruction revealed ADP bound at the $\rho^{P167L}$ nucleotide-binding pockets, suggesting that $\rho^{P167L}$ hydrolyzed ATPγS at least partially during sample preparation. Indeed, hydrolysis of ATPγS has also been observed before in other systems[35].

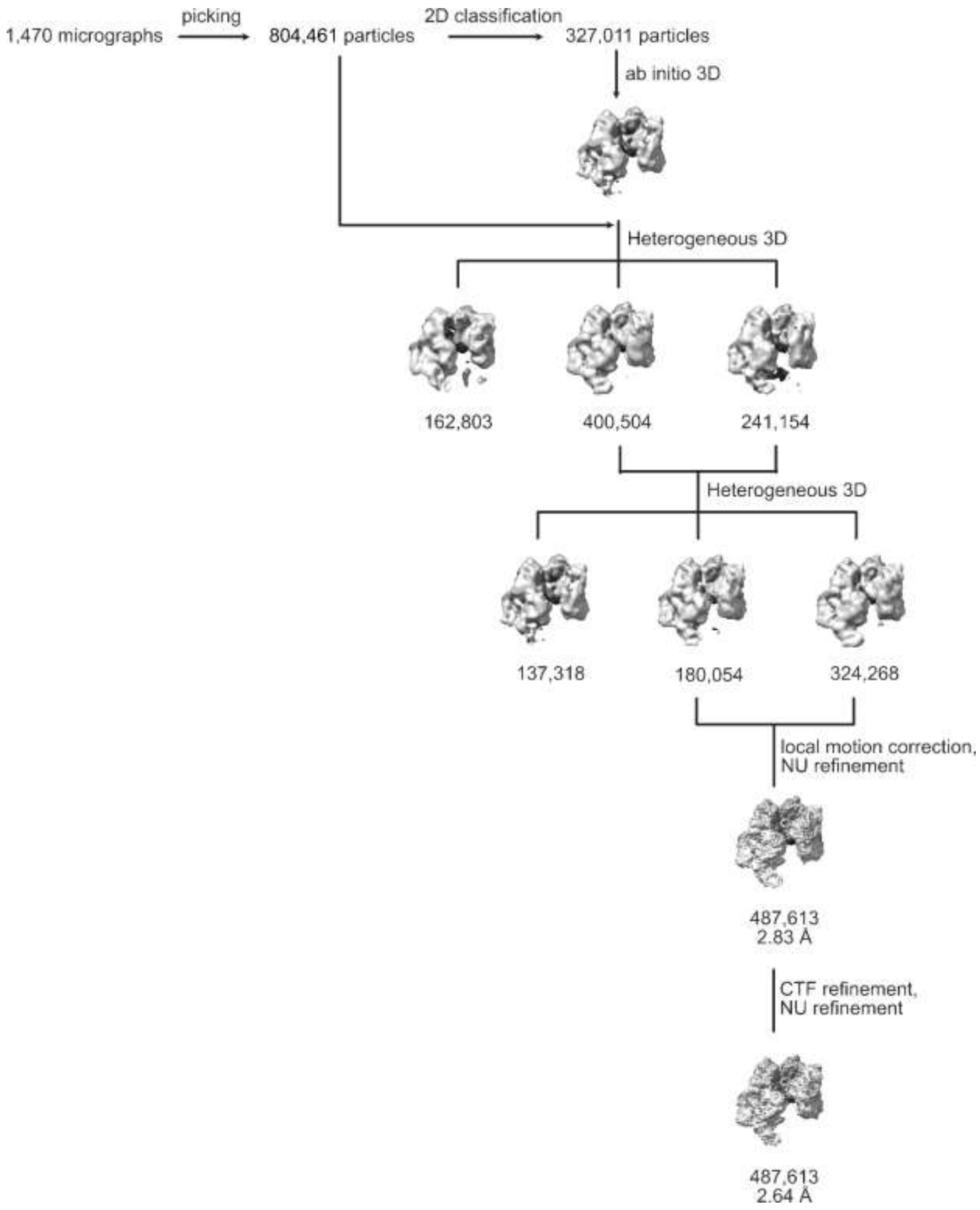

1,470 micrographs
picking
804,461 particles
2D classification
327,011 particles
ab initio 3D
Heterogeneous 3D
162,803
400,504
241,154
Heterogeneous 3D
137,318
180,054
324,268
local motion correction,
NU refinement
487,613
2.83 Å
CTF refinement,
NU refinement
487,613
2.64 Å

**Supplementary Fig. 11: $\rho^{P167L}$-ATPγS cryoEM data refinement.**

804,461 particles were picked from 1,470 micrographs and subjected to reference-free 2D classification. 327,011 particle images were selected and used for ab initio 3D reconstruction, followed by iterative cycles of heterogeneous refinement including all picked particle images. 487,613 particles were selected and subjected to local motion correction followed by NU refinement. Final NU refinement after CTF refinement yielded a reconstruction at 2.64 Å resolution.

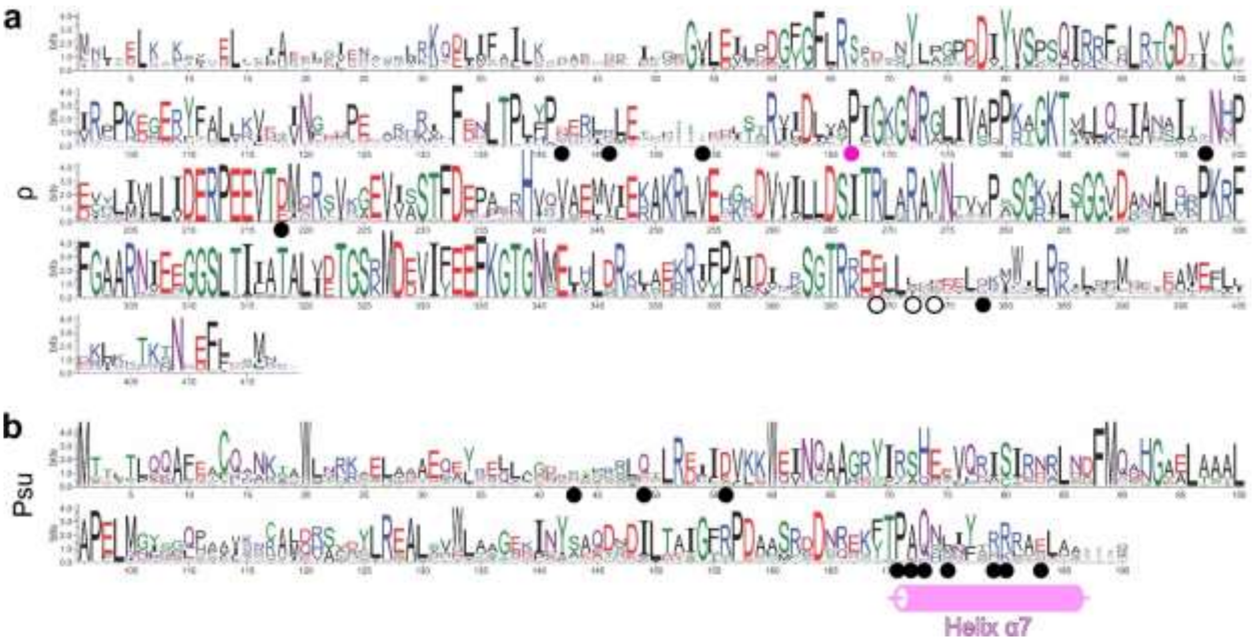


**Supplementary Fig. 12: ρ and Psu sequence conservation.**

**a**, Conservation pattern of ρ based on a multiple sequence alignment of ρ proteins from representatives of the entire Bacteria kingdom.[36] The universally conserved P167 residue is marked by a magenta sphere. Residues exhibiting side chain interactions between *E. coli* ρ and phage P4 Psu are marked by black spheres; residues exhibiting backbone interactions are marked by white spheres.

**b**, Conservation pattern of Psu proteins mined in this study (Supplementary Data 1a).

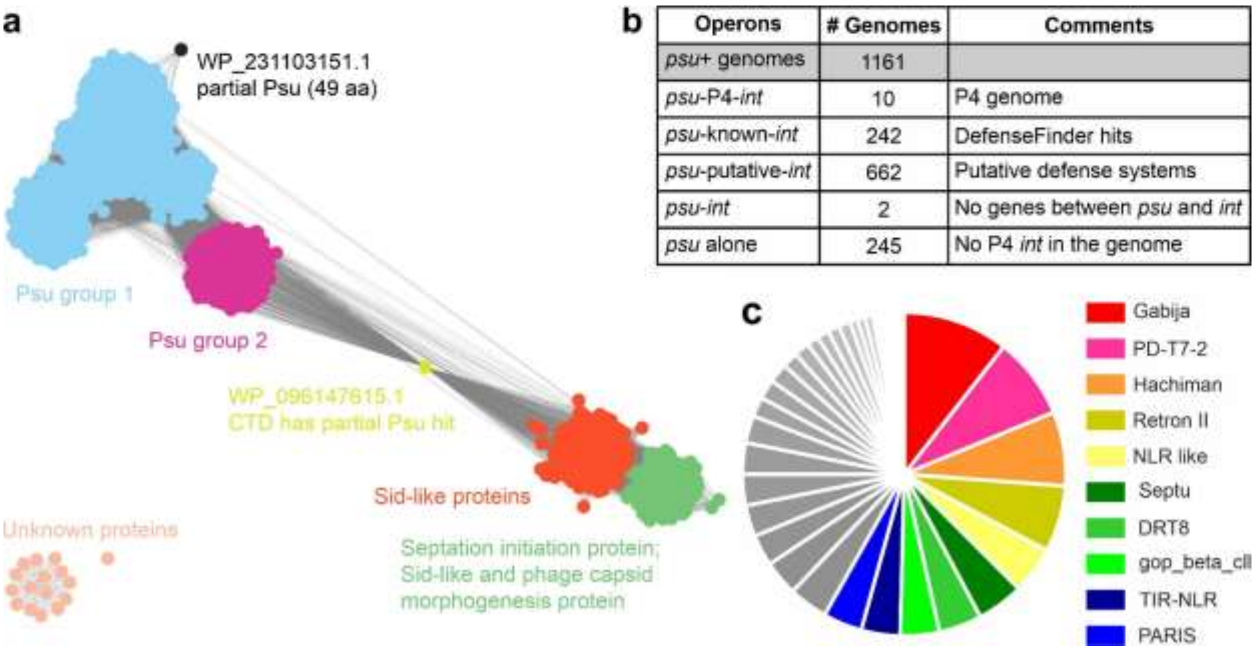


| Operons | # Genomes | Comments |
|---|---|---|
| *psu*+ genomes | 1161 | |
| *psu*-P4-*int* | 10 | P4 genome |
| *psu*-known-*int* | 242 | DefenseFinder hits |
| *psu*-putative-*int* | 662 | Putative defense systems |
| *psu*-*int* | 2 | No genes between *psu* and *int* |
| *psu* alone | 245 | No P4 *int* in the genome |

**Supplementary Fig. 13: Psu-linked phage defense systems.**

**a**, Psu homologs form two groups. See Methods for details.

**b**, Analysis of *psu*$^{+}$ genomes reveals many potential defense systems. See Supplementary Data 1b-e for genomic context details. We note that the absence of the *int* gene could be due to incomplete genome assembly in some cases and ~60 % *psu* genes in the "*psu* alone" group are located at the terminus of linearized genomes.

**c**, The 36 classes of *psu*-associated defense systems identified by DefenseFinder are shown in a pie chart, with the ten most abundant defense systems indicated in color; see Supplementary Data 1c for a complete list.

## Supplementary References


1. Ding, F., Prutzman, K.C., Campbell, S.L. & Dokholyan, N.V. Topological determinants of protein domain swapping. *Structure* **14**, 5-14 (2006).
2. Karreman, C. & de Waard, A. Cloning and complete nucleotide sequences of the type II restriction-modification genes of Salmonella infantis. *J Bacteriol* **170**, 2527-2532 (1988).
3. Kita, K. et al. Evidence of horizontal transfer of the EcoO109I restriction-modification gene to Escherichia coli chromosomal DNA. *J Bacteriol* **181**, 6822-6827 (1999).
4. Lee, K.F., Shaw, P.C., Picone, S.J., Wilson, G.G. & Lunnen, K.D. Sequence comparison of the EcoHK31I and EaeI restriction-modification systems suggests an intergenic transfer of genetic material. *Biol Chem* **379**, 437-441 (1998).
5. Moura de Sousa, J.A. & Rocha, E.P.C. To catch a hijacker: abundance, evolution and genetic diversity of P4-like bacteriophage satellites. *Philos Trans R Soc Lond B Biol Sci* **377**, 20200475 (2022).
6. Rousset, F. et al. Phages and their satellites encode hotspots of antiviral systems. *Cell Host Microbe* **30**, 740-753 e745 (2022).
7. Vassallo, C.N., Doering, C.R., Littlehale, M.L., Teodoro, G.I.C. & Laub, M.T. A functional selection reveals previously undetected anti-phage defence systems in the E. coli pangenome. *Nat Microbiol* **7**, 1568-1579 (2022).
8. Lagos, R., Jiang, R.Z., Kim, S. & Goldstein, R. Rho-dependent transcription termination of a bacterial operon is antagonized by an extrachromosomal gene product. *Proc Natl Acad Sci U S A* **83**, 9561-9565 (1986).
9. Lindqvist, B.H., Deho, G. & Calendar, R. Mechanisms of genome propagation and helper exploitation by satellite phage P4. *Microbiol Rev* **57**, 683-702 (1993).
10. Tesson, F. et al. Systematic and quantitative view of the antiviral arsenal of prokaryotes. *Nat Commun* **13**, 2561 (2022).
11. Doron, S. et al. Systematic discovery of antiphage defense systems in the microbial pangenome. *Science* **359**(2018).
12. Gao, L. et al. Diverse enzymatic activities mediate antiviral immunity in prokaryotes. *Science* **369**, 1077-1084 (2020).
13. Millman, A. et al. An expanded arsenal of immune systems that protect bacteria from phages. *Cell Host Microbe* **30**, 1556-1569 e1555 (2022).
14. Garb, J. et al. Multiple phage resistance systems inhibit infection via SIR2-dependent NAD(+) depletion. *Nat Microbiol* **7**, 1849-1856 (2022).
15. Zaremba, M. et al. Short prokaryotic Argonautes provide defence against incoming mobile genetic elements through NAD(+) depletion. *Nat Microbiol* **7**, 1857-1869 (2022).
16. Ofir, G. et al. Antiviral activity of bacterial TIR domains via immune signalling molecules. *Nature* **600**, 116-120 (2021).
17. Santangelo, T.J. & Artsimovitch, I. Termination and antitermination: RNA polymerase runs a stop sign. *Nat Rev Microbiol* **9**, 319-329 (2011).
18. Hu, K. & Artsimovitch, I. A Screen for rfaH Suppressors Reveals a Key Role for a Connector Region of Termination Factor Rho. *mBio* **8**(2017).

19. Linderoth, N.A., Tang, G. & Calendar, R. In vivo and in vitro evidence for an anti-Rho activity induced by the phage P4 polarity suppressor protein Psu. *Virology* **227**, 131-141 (1997).

20. Sunshine, M. & Six, E. Relief of P2 bacteriophage amber mutant polarity by the satellite bacteriophage P4. *J Mol Biol* **106**, 673-682 (1976).

21. Bossi, L. et al. NusG prevents transcriptional invasion of H-NS-silenced genes. *PLoS Genet* **15**, e1008425 (2019).

22. Williams, C.J. et al. MolProbity: More and better reference data for improved all-atom structure validation. *Protein Sci* **27**, 293-315 (2018).

23. Pani, B., Ranjan, A. & Sen, R. Interaction surface of bacteriophage P4 protein Psu required for complex formation with the transcription terminator Rho. *J Mol Biol* **389**, 647-660 (2009).

24. Sauer, B., Ow, D., Ling, L. & Calendar, R. Mutants of satellite bacteriophage P4 that are defective in the suppression of transcriptional polarity. *J Mol Biol* **145**, 29-46 (1981).

25. Isaksen, M.L., Rishovd, S.T., Calendar, R. & Lindqvist, B.H. The polarity suppression factor of bacteriophage P4 is also a decoration protein of the P4 capsid. *Virology* **188**, 831-839 (1992).

26. Pani, B. et al. Mechanism of inhibition of Rho-dependent transcription termination by bacteriophage P4 protein Psu. *J Biol Chem* **281**, 26491-26500 (2006).

27. Ranjan, A., Sharma, S., Banerjee, R., Sen, U. & Sen, R. Structural and mechanistic basis of anti-termination of Rho-dependent transcription termination by bacteriophage P4 capsid protein Psu. *Nucleic Acids Res* **41**, 6839-6856 (2013).

28. Krupp, F. et al. Structural Basis for the Action of an All-Purpose Transcription Anti-Termination Factor. *Mol Cell* **74**, 143-157 (2019).

29. Said, N. et al. Steps toward translocation-independent RNA polymerase inactivation by terminator ATPase rho. *Science* **371**(2021).

30. Muteeb, G., Dey, D., Mishra, S. & Sen, R. A multipronged strategy of an anti-terminator protein to overcome Rho-dependent transcription termination. *Nucleic Acids Res* **40**, 11213-11228 (2012).

31. Valabhoju, V., Agrawal, S. & Sen, R. Molecular Basis of NusG-mediated Regulation of Rho-dependent Transcription Termination in Bacteria. *J Biol Chem* **291**, 22386-22403 (2016).

32. Ghosh, G., Sharma, P.V., Kumar, A., Jain, S. & Sen, R. Design of novel peptide inhibitors against the conserved bacterial transcription terminator, Rho. *J Biol Chem* **296**, 100653 (2021).

33. Tan, Y.Z. et al. Addressing preferred specimen orientation in single-particle cryo-EM through tilting. *Nat Methods* **14**, 793-796 (2017).

34. Baldwin, P.R. & Lyumkis, D. Non-uniformity of projection distributions attenuates resolution in Cryo-EM. *Prog Biophys Mol Biol* **150**, 160-183 (2020).

35. Peck, M.L. & Herschlag, D. Adenosine 5'-O-(3-thio)triphosphate (ATPgammaS) is a substrate for the nucleotide hydrolysis and RNA unwinding activities of eukaryotic translation initiation factor eIF4A. *RNA* **9**, 1180-1187 (2003).

36. Wang, B. et al. Transcription termination factor rho polymerizes under stress. *bioRxiv* (2023).

# Source Data

## Supplementary Fig. 1a

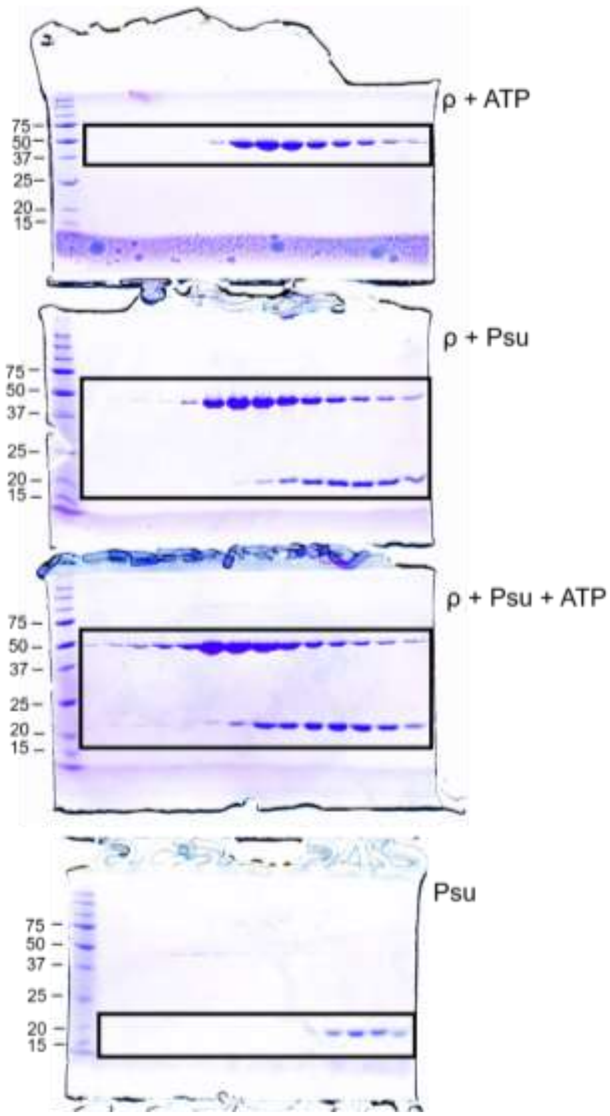


## Supplementary Fig. 1c

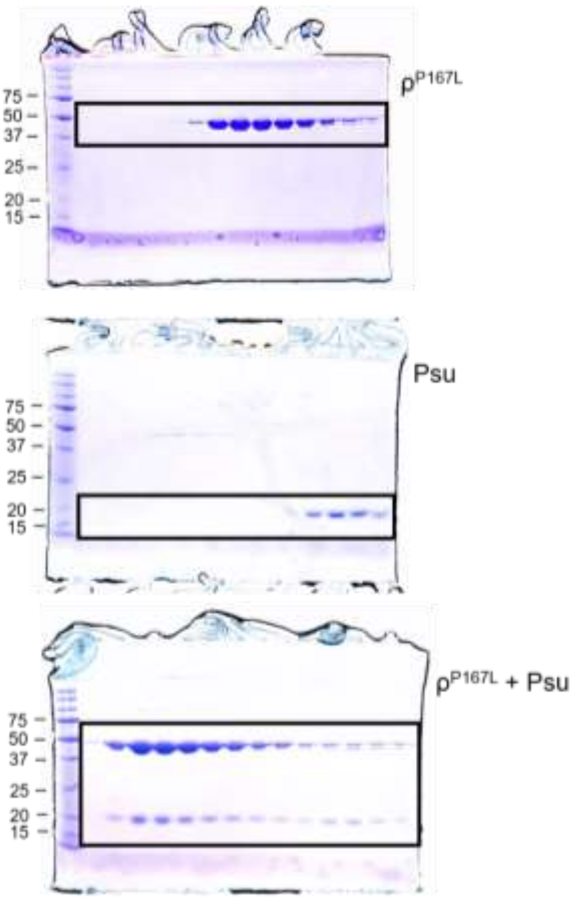

Supplement: Supplementary file 1 — Supplementary Information [file 41467_2025_55897_MOESM1_ESM.pdf]
