# Supplementary material for: The Psu protein of phage satellite P4 inhibits transcription termination factor ρ by forced hyper-oligomerization
Source: Nat Commun. 2025 Jan 9;16:550. doi: 10.1038/s41467-025-55897-9 (PMC11718236; doi:10.1038/s41467-025-55897-9)
Supplement: Supplementary file 2 — Description of Additional Supplementary Files [file 41467_2025_55897_MOESM2_ESM.pdf]

Description of Additional Supplementary Files

File name: Supplementary Data 1
Description: The ρ and Psu in silico analysis dataset
a, All of the Psu NCBI IDs.
b, *psu*-P4-*int* loci. P4 prophage genomes.
c, *psu*-known-*int* loci. Phage defense systems found by DefenseFinder.
d, *psu*-putative-*int* loci. Putative denfense systems located between *psu* and *int.*
e, *psu* alone. Loci without *int.*
f, Pfam-A database scanning for putative defense systems.
g, Gene number counts of the Pfam-A hits.
